# Does the presence of systemic artery–pulmonary circulation shunt during bronchial arterial embolization increase the recurrence of noncancer-related hemoptysis? A retrospective cohort study

**DOI:** 10.1186/s12931-023-02427-0

**Published:** 2023-05-02

**Authors:** Hai-Tao Yan, Guang-Dong Lu, Jin Liu, Sheng Liu, Hai-Bin Shi, Chun-Gao Zhou, Qing-Quan Zu

**Affiliations:** 1 Department of Interventional Radiology, The First Affiliated Hospital with Nanjing Medical University, No. 300 Guangzhou Road, Nanjing, 210029 China; 2 Department of Clinical Medicine Research Institution, The First Affiliated Hospital with Nanjing Medical University, Nanjing, 210029 China

**Keywords:** Cohort studies, Hemoptysis, Embolization, Therapeutic, Pulmonary circulation

## Abstract

**Background:**

The presence of systemic artery–pulmonary circulation shunt (SPS) during the bronchial arterial embolization (BAE) procedure, has been inferred to be a potential risk factor for recurrence. The aim of this study is to reveal the impact of SPS on the recurrence of noncancer-related hemoptysis after BAE.

**Methods:**

In this study, 134 patients with SPS (SPS-present group) and 192 patients without SPS (SPS-absent group) who underwent BAE for noncancer-related hemoptysis from January 2015 to December 2020 were compared. Four different Cox proportional hazards regression models were used to clarify the impact of SPSs on hemoptysis recurrence after BAE.

**Results:**

During the median follow-up time of 39.8 months, recurrence occurred in 75 (23.0%) patients, including 51 (38.1%) in the SPS-present group and 24 (12.5%) in the SPS-absent group. The 1-month, 1-year, 2-year, 3-year and 5-year hemoptysis-free survival rates in the SPS-present and SPS-absent groups were 91.8%, 79.7%, 70.6%, 62.3%, and 52.6% and 97.9%, 94.7%, 89.0%, 87.1%, and 82.3%, respectively (P < 0.001). The adjusted hazard ratios of SPSs in the four models were 3.37 [95% confidence intervals (CI), 2.07–5.47, P < 0.001 in model 1], 1.96 (95% CI, 1.11–3.49, P = 0.021 in model 2), 2.29 (95% CI, 1.34–3.92, P = 0.002 in model 3), and 2.39 (95% CI, 1.44–3.97, P = 0.001 in model 4).

**Conclusions:**

The presence of SPS during BAE increases the recurrence probability of noncancer-related hemoptysis after BAE.

## Introduction

Hemoptysis is a common symptom of respiratory diseases and can be life-threatening due to the risk of asphyxia and acute blood loss. In recent decades, bronchial arterial embolization (BAE) has been recognized as a minimally invasive and effective method for controlling hemoptysis [1]. However, recurrence of noncancer-related hemoptysis after BAE is still common. The early (≤ 1 month) recurrence rate has remained below 10%, while the long-term cumulative recurrence rate is as high as 30% [2–5]. Among patients with relapse, 40–60% require repeat embolization or lobectomy or experience death [3, 6, 7].

The definitive risk factors for recurrence after BAE include heavy smoking, lung destruction, aspergillomas, and culprit vessels from nonbronchial systemic arteries (NBSAs) [3, 6–8]. In addition, another arteriography-specific parameter during the BAE procedure, the presence of systemic artery–pulmonary circulation shunt (SPS), has been inferred to be a potential risk factor for recurrence, although this has remained controversial [2, 3, 6, 8–11]. There is a vascular network between the systemic and pulmonary circulatory circuits at both the capillary and precapillary levels under natural physiologic and anatomic conditions [12]. Long-course pulmonary inflammation induces hypertrophy of the systemic vessels and then amplifies these communicating vessels as substitute shunts.

These shunts open pathologically and are prone to rupture under chronic inflammation and systemic arterial pressure [13]. In this context, an SPS for hemodynamic regulation is applied as a compensatory alteration in ~ 30% of hemoptysis patients [14, 15]. However, the effect of SPSs has received relatively little attention in previous studies, and some results have shown the negative predictive value of SPSs for recurrence [6, 11, 16]. A previous study revealed that the incidence of same-vessel recanalization in patients with SPSs (80.0%) seemed to be higher than that in patients without SPSs (30.8%) [3], which may also provide clues regarding the mechanism of recurrence. Given these findings, clarification of whether the presence of SPS during BAE is an independent risk factor for recurrence after embolization is necessary and may initiate the exploration of further treatment strategies. We hypothesized that the presence of shunt would increase recurrence of noncancer-related hemoptysis after BAE.

Thus, we conducted a retrospective cohort study to investigate the association between the presence of SPS and recurrence of noncancer-related hemoptysis after endovascular treatment.

## Materials and methods

The research protocol of this retrospective study was discussed and approved by the local institutional ethics review board, and the requirement for informed consent was waived due to its retrospective nature. All procedures performed in this study were in accordance with the ethical standards of the institutional and/or national research committee and with the 1964 Helsinki Declaration and its later amendments or comparable ethical standards. The data analyzed in this study is available upon reasonable request from the corresponding author.

### Study participants

**Fig. 1:**
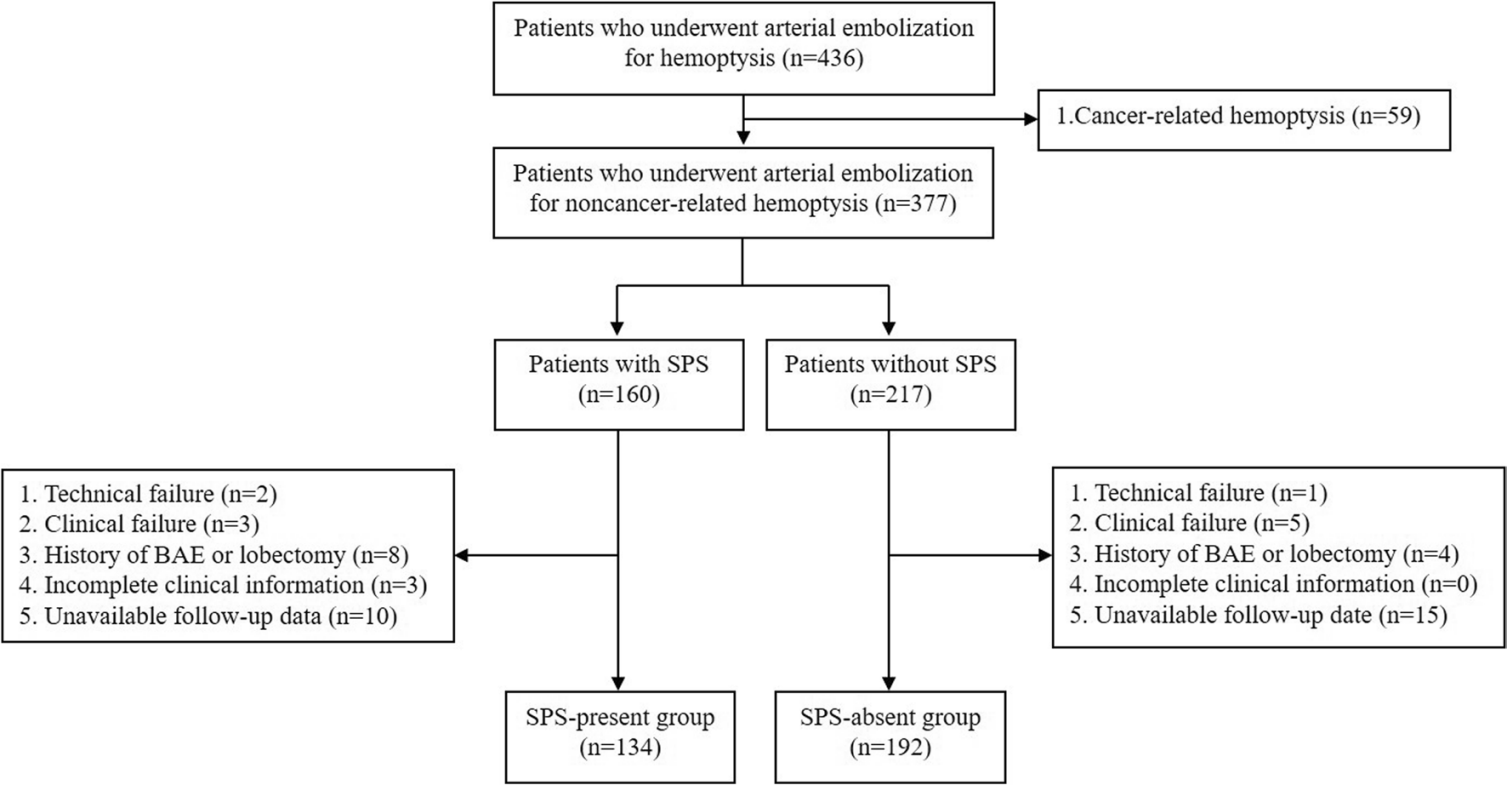
Flowchart of patient enrollment

**Fig. 2:**
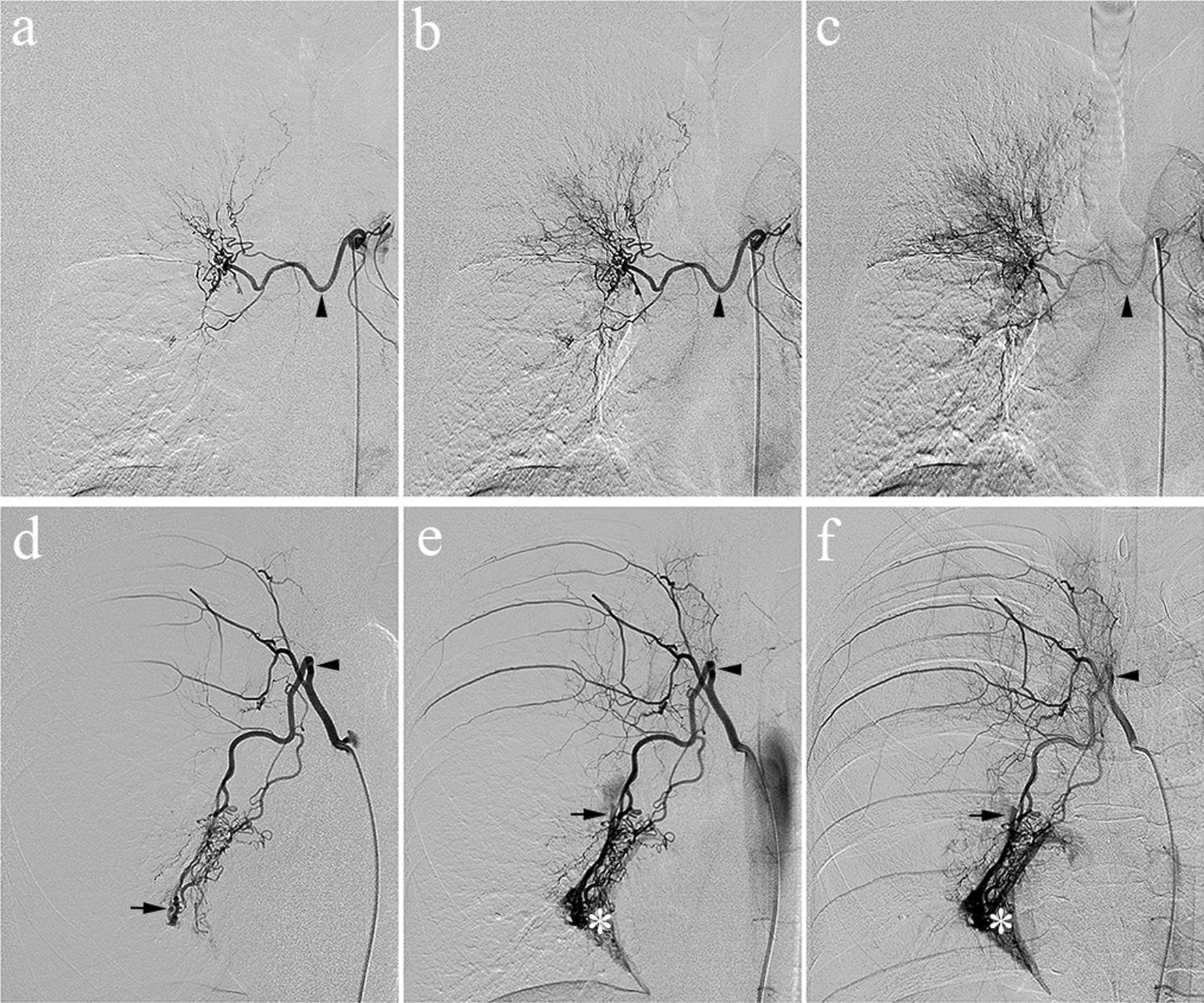
Representative images of patients with/without bronchial artery–pulmonary circulation shunt on angiography. 62-year-old man with cryptogenic hemoptysis (**a**–**c**). The very early (**a**), early (**b**), and late (**c**) arterial phases on angiography of the culprit bronchial artery (black arrowhead) without the presence of a shunt. 31-year-old woman with bronchiectasis (**d**–**f**). Angiography of the culprit bronchial artery (black arrowhead) revealed that the bronchial artery–pulmonary circulation shunt (black arrow) was opacified in the very early phase arterial phase (**d**), which remained opacified in the early and late arterial phases accompanied by lung parenchyma staining (*) (**e**–**f**)

We queried the baseline information, preoperative computed tomography (CT) and angiographic data of 436 consecutive adult patients who underwent arterial embolization for hemoptysis at our institution between January 2015 and December 2020. The exclusion criteria were as follows: (1) cancer-related hemoptysis (n = 59); (2) technical failure (n = 3); (3) clinical failure (n = 8); (4) history of BAE or lobectomy (n = 12); (5) incomplete clinical information (n = 3); and (6) unavailable follow-up date (n = 25). Ultimately, 134 patients were enrolled in the SPS-present group, while 192 patients were enrolled in the SPS-absent group. Figure 1 shows the patient enrollment flowchart. The presence of SPS was identified by the presence of feeding arteries (bronchial arteries and NBSAs) and pathological communicating and drainage vessels (Fig. 2) on dynamic angiography by two doctors independently; the decision was discussed with a senior doctor, and an agreement was reached, ensuring the reliability of the assessment.

### Covariates

The covariates in the present study included baseline information (age, sex, underlying lung disease, smoking, hypertension, duration and volume of hemoptysis), preoperative CT findings (number of affected lobes, presence of pleural thickening, and lung destruction), and angiographic data (number and diameter of culprit bronchial arteries, presence of NBSAs, and embolization materials). Hemoptysis severity was graded and classified into three levels according to the volume of hemoptysis: mild (< 100 ml/d), moderate (100–300 ml/d), and massive (≥ 300 ml/d) [17]. Eight patients underwent bronchoscopy, CT and CT angiography (CTA) were performed for every patient during hospitalization. They could show underlying lung disease, locate the bleeding source, identify origins and courses of bronchial arteries and NBSAs, which provide high possibility for clinical success after BAE. The underlying lung disease included bronchiectasis, tuberculosis sequelae, chronic pneumonia, and cryptogenic hemoptysis. Cryptogenic hemoptysis indicated a lack of specific parenchymal or vascular abnormalities in the lung, as noted on preoperative CTA [18]. Pleural thickening > 3 mm was diagnosed as pathological [19]. Irreversible parenchymal destruction characterized by diffuse adhesions and large cavities was defined as lung destruction [20].

### Arterial embolization procedures

All patients received standard medical care, including vital sign monitoring, hypoxemia correction, and hemostasis. The angiographic machines used included the Artis Zeego Digital Subtraction Angiography (DSA) (Siemens, Germany) and UNIQ FD20 DSA (PHILIPS, Netherlands) devices. The BAE procedure was performed by two interventional physicians with 8 and 10 years of experience. Before the procedure, they reviewed CT and CTA images to observe anatomy of the culprit artery and searched suspicious NBSAs according to the location of lung lesion, in particular, inferior phrenic arteries for inferior lobe lesion, internal mammary or esophageal arteries for medial lung lesion and superior thoracic arteries for superior lobe lesion, lateral thoracic arteries for lateral lung lesion, etc.. All procedures were performed via the femoral artery approach. A 5-F angiographic catheter (Cobra, RLG, MIK; Cook, USA) was inserted into suspicious culprit vessels (bronchial and nonbronchial arteries) to perform angiography with a total volume of 6–10 ml contrast agent (iodixanol 320 mgI/ml, GE Healthcare, United States or iopromide 370 mgI/ml, BAYER, Germany) injected at a speed of 1.5–2.0 ml/s. The abnormal angiographic findings included contrast agent extravasation, hypertrophic and tortuous vessels, hypervascularity, and presence of an SPS [21]. Then, a microcatheter (2.7F; Terumo, Tokyo, Japan; or 2.4F; Merit Maestro, Utah, USA) was advanced as distally as possible to avoid ectopic embolization. The embolic materials were polyvinyl alcohol (PVA) particles (300–500 μm; Cook, USA), microspheres (500–700 μm; Merit Maestro, Utah, USA), and gelatin sponge particles (350–560 μm; Hangzhou Alicon Pharmaceutical Co., Ltd., Zhejiang, China). Microcoils (Cook, USA) were used for large vessel diameters or aneurysmal dilatation of culprit vessels after initial embolization with particles. The endpoint of embolization was complete occlusion of all the culprit arteries.

### Follow-up and recurrence

Technical success was reflected by immediately successful embolization of all culprit vessels; otherwise, the procedure was considered a technical failure [22]. Clinical success was defined as the resolution of hemoptysis within 24 h after the procedure; otherwise, the procedure was defined as a clinical failure [22]. Severe procedure-related complications resulted in prolonged hospitalization, advanced care, unscheduled admission for therapy after discharge, permanent sequelae or death; all other complications were considered minor complications [23].

After discharge, follow-up evaluations in an outpatient clinic were scheduled at approximately 1–3 months. Subsequently, the patient was followed up by telephone interview approximately every six months for the first two years. If there was no recurrence, the follow-up calls were reduced to once a year. The follow-up assessed the general condition of the patient, queried details of hemoptysis recurrence (date of relapse, volume of hemoptysis, and management details) and provided healthy lifestyle education, including on smoking cessation, precautions according to the season of the year, abdominal respiration, and controlling pulmonary infections with low-dose macrolide therapy. Recurrence was defined as relapse with a hemoptysis volume ≥ 30 ml per day, the need for repeated BAE or lobectomy, or death due to recurrent hemoptysis after clinical success [11]. According to the angiographic findings during repeated BAE, the cause of the recurrence was also documented; these causes included missed culprit arteries, recanalization and new collateral arteries. The end date of follow-up was May 2021 or the date of death.

### Statistical analysis

All data analyses were performed with the Statistical Package for the Social Sciences (SPSS, version 22.0, Armonk, NY, USA). Patients with complete follow-up information were included in the final analysis. Continuous variables are described as the means and standard deviations (SD), and categorical variables are expressed as numbers (%). We compared the baseline characteristics of patients in the SPS-absent and SPS-present groups. Categorical variables were compared with the χ^2^ test or Fisher’s exact test. Continuous variables were compared with the t test or the Wilcoxon test. Cumulative hemoptysis-free curves were estimated by the Kaplan–Meier method. The impact of SPSs on recurrence was assessed by four different Cox proportional hazards regression models. Model 1 was a univariate Cox proportional hazards regression model. Model 2 was the full model that integrated all potential factors. Then, multivariate Cox analysis was performed to identify the statistically significant variables (P < 0.1) in Model 2, which was described as Model 3. In Model 4, the presence of SPS was the only factor maintained in the model, and the other variables were screened in a forward stepwise manner. A P value < 0.05 was considered to indicate statistical significance. A forest plot was generated with GraphPad Software (Prism 8.0.1, San Diego, California) according to the hazard ratio (HR) and 95% confidence interval (CI) of different models.

## Results

### Characteristics of the study cohort

The baseline characteristics of the study cohort are provided in Table 1. The SPS-present group had more female patients (P = 0.001), a different composition of underlying lung disease (P < 0.001), a longer duration of hemoptysis (P < 0.001), more affected lobes (P < 0.001), culprit arteries with larger diameters (P < 0.001), and a higher incidence of pleural thickening (P < 0.001), lung destruction (P = 0.001) and culprit NBSAs (P < 0.001) than the SPS-absent group. The other variables did not show significant differences between groups.

**Table 1:** Baseline characteristics of the patients

Parameters	All patients (n = 326)	SPS-absent group (n = 192)	SPS-present group (n = 134)	*P*-value
Age (years)	59.2 ± 13.1	58.7 ± 13.3	59.9 ± 12.9	0.430
Sex				0.001
Female	95 (29.1%)	43 (22.4%)	52 (38.8%)	
Male	231 (70.9%)	149 (77.6%)	82 (61.2%)	
Duration of hemoptysis (months)				< 0.001
> 6	122 (37.4%)	55 (28.6%)	67 (50%)	
≤ 6	204 (62.6%)	137 (71.4%)	67 (50%)	
Underlying lung disease				< 0.001
Bronchiectasis	175 (53.7%)	93 (48.4%)	82 (61.2%)	
Tuberculosis sequela	88 (27.0%)	43 (22.4%)	45 (33.6%)	
Chronic pneumonia	37 (11.3%)	32 (16.7%)	5 (3.7%)	
Cryptogenic hemoptysis	26 (8.0%)	24 (12.5%)	2 (1.5%)	
Volume of hemoptysis (ml/d)				0.255
< 100	127 (39.0%)	79 (41.1%)	48 (35.8%)	
100–300	135 (41.4%)	81 (42.2%)	54 (40.3%)	
≥ 300	64 (19.6%)	32 (16.7%)	32 (23.9%)	
Smoking	102 (29.1%)	65 (33.9%)	37 (27.6%)	0.232
Hypertension	93 (28.5%)	58 (30.2%)	35 (26.1%)	0.421
Disease extent (number of affected lobes)	2.1 ± 1.2	1.9 ± 1.2	2.5 ± 1.1	< 0.001
Presence of pleural thickening	182 (55.8%)	82 (42.7%)	100 (74.6%)	< 0.001
Lung destruction	21 (6.4%)	5 (2.6%)	16 (11.9%)	0.001
Number of culprit bronchial arteries	2.3 ± 1.0	2.2 ± 1.0	2.3 ± 1.0	0.677
Diameter of culprit bronchial arteries (mm)	2.8 ± 1.1	2.5 ± 0.9	3.1 ± 1.2	< 0.001
Presence of culprit NBSAs	82 (25.2%)	25 (13.0%)	57 (42.5%)	< 0.001
Embolization materials				0.273
PVA	261 (80.0%)	148 (77.1%)	113 (84.3%)	
Microsphere	56 (17.2%)	38 (19.8%)	18 (13.5%)	
Gelatin sponge	9 (2.8%)	6 (3.1%)	3 (2.2%)	

*NBSAs* nonbronchial systemic arteries, *PVA* polyvinyl alcohol, *SPS* systemic artery–pulmonary circulation shunt

### Arterial embolization and complications

In total, we embolized 832 culprit arteries, including 733 bronchial arteries (401 in the right lung, 332 in the left lung) and 99 NBSAs, with an average of 2.6 arteries per patient. Culprit NBSAs were identified in 57 (42.5%) patients in the SPS-present group and 25 (13.0%) patients in the SPS-absent group. There were no significant differences in the number of culprit bronchial arteries and embolization materials (P = 0.677 and P = 0.273, respectively). Microcoils were used to treat high-level shunts in 10 patients (bronchial artery: 9 patients; NBSAs: 1 patient) and for aneurysmal dilatation of culprit arteries in 6 patients (bronchial artery: 5 patients; NBSAs: 1 patient).

Minor complications were observed in 60 patients, including chest or shoulder pain (n = 32), fever (n = 20), vomiting (n = 4), abdominal pain (n = 3) and puncture site discomfort (n = 1), all of which were relieved by conservative treatment. One patient in the SPS-present group suffered cerebral infarction after BAE with 300–500 μm PVA. There were no significant differences in either the minor or major complication rates between the two groups: 19.4% (26/134) and 0.7% (1/134) for the SPS-present group and 17.7% (34/192) and 0 for the SPS-absent group (P = 0.698 and P = 0.856, respectively).

### Shunt and recurrence

**Fig. 3:**
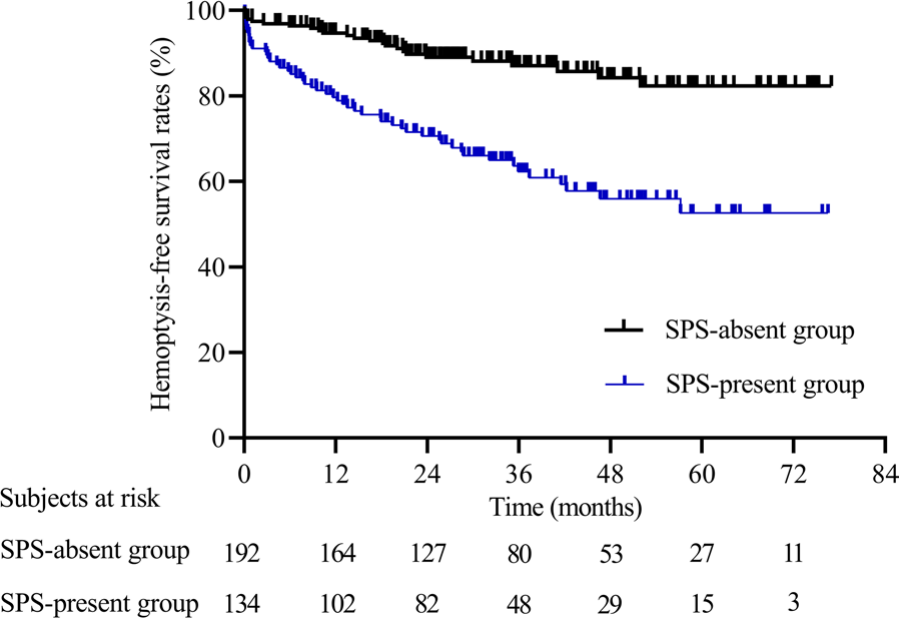
Kaplan–Meier curves for recurrence-free time in the SPS-absent group and SPS-present group (P < 0.001)

During the median follow-up time of 39.8 months, recurrence was observed in 75 (23.0%) patients, 51 (38.1%) of whom were in the SPS-present group and 24 (12.5%) of whom were in the SPS-absent group. There was no difference in the median follow-up time between the two groups (38.6 vs. 42.6 months, P = 0.271). The number of recurrence events in the SPS-present group, there were 27, 11, 8, 4 and 1 recurrence events in the first, second, third, fourth and fifth years, while the corresponding numbers in the SPS-absent group were 10, 9, 2, 2 and 1. The 1-month, 1-year, 2-year, 3-year and 5-year hemoptysis-free survival rates of 91.8%, 79.7%, 70.6%, 62.3%, and 52.6% in the SPS-present group were significantly lower than the rates of 97.9%, 94.7%, 89.0%, 87.1%, and 82.3% in the SPS-absent group (P < 0.001) (Fig. 3). In the SPS-present group, 24 patients underwent repeated BAE, 2 patients underwent segmentectomy, 19 patients received medical therapy for recurrent hemoptysis, and 6 patients died due to recurrent hemoptysis; in the SPS-absent group, 8 patients underwent repeated BAE, and 16 patients received medical therapy. According to the angiography results from repeated BAE, the causes of relapse included missed culprit arteries (4 in the SPS-present group; 2 in the SPS-absent group, P = 0.601) for early recurrence, new collateral artery formation (7 in the SPS-present group; 5 in the SPS-absent group, P = 0.092) and recanalization (13 in the SPS-present group; 1 in the SPS-absent group, P = 0.040) for later recurrence.

**Fig. 4:**
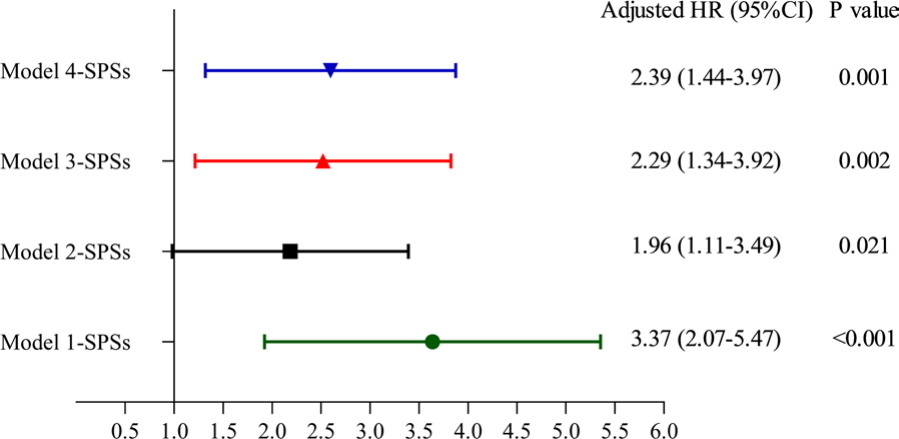
The forest plot was constructed according to the adjusted HR (95% CI) and P values of SPSs in four different Cox proportional hazards regression models

The adjusted HRs of SPSs in the four models were 3.37 (95% CI, 2.07–5.47, P < 0.001 in model 1), 1.96 (95% CI, 1.11–3.49, P = 0.021 in model 2), 2.29 (95% CI, 1.34–3.92, P = 0.002 in model 3), and 2.39 (95% CI, 1.44–3.97, P = 0.001 in model 4) (Table 2). Based on these results, we generated a forest plot (Fig. 4).

**Table 2:** Analyses of the relationship between SPSs and recurrence after BAE based on different statistical models

Model	Parameters	Level	HR (95% CI)	*P* value
Model 1	SPSs	Present	3.37 (2.07–5.47)	< 0.001
		Absent	Reference	
Model 2	SPSs	Present	1.96 (1.11–3.49)	0.021
		Absent	Reference	
	Age	–	1.00 (0.98–1.02)	0.739
	Sex	Female	1.42 (0.79–2.54)	0.244
		Male	Reference	
	Duration of hemoptysis	> 6 months	1.54 (0.92–2.57)	0.098
		≤ 6 months	Reference	
	Underlying lung disease			0.823
		Bronchiectasis	0.91 (0.18–4.56)	0.909
		Tuberculosis sequela	0.99 (0.19–5.03)	0.989
		Chronic pneumonia	0.47 (0.06–3.74)	0.478
		Cryptogenic hemoptysis	Reference	
	Volume of hemoptysis			0.672
		≥ 300 ml/d	1.11 (0.58–2.12)	0.744
		100–300 ml/d	1.27 (0.75–2.16)	0.376
		< 100 ml/d	Reference	
	Smoking	Present	1.03 (0.56–1.89)	0.924
		Absent	Reference	
	Hypertension	Present	0.87 (0.48–1.60)	0.663
		Absent	Reference	
	Disease extent (number of affected lobes)	–	1.32 (0.99–1.75)	0.058
	Presence of pleural thickening	Present	1.04 (0.55–1.97)	0.901
		Absent	Reference	
	Lung destruction	Present	1.77 (0.80–3.88)	0.157
		Absent	Reference	
	Number of culprit BAs	–	0.89 (0.69–1.14)	0.355
	Presence of culprit NBSAs	Present	1.58 (0.92–2.71)	0.098
		Absent	Reference	
	Embolization materials			0.443
		Gelatin sponge	2.14 (0.66–6.93)	0.206
		Microsphere	0.97 (0.45–2.09)	0.938
		PVA	Reference	
Model 3	SPSs	Present	2.29 (1.34–3.92)	0.002
		Absent	Reference	
	Duration of hemoptysis	> 6 months	2.00 (1.24–3.23)	0.004
		≤ 6 months	Reference	
	Presence of culprit NBSAs	Present	1.79 (1.09–2.92)	0.021
		Absent	Reference	
Model 4	SPSs	Present	2.39 (1.44–3.97)	0.001
		Absent	Reference	
	Duration of hemoptysis	> 6 months	1.78 (1.10–2.88)	0.020
		≤ 6 months	Reference	
	Disease extent (number of affected lobes)	–	1.30 (1.02–1.64)	0.033
	Lung destruction	Present	2.07 (1.03–4.18)	0.042
		Absent	Reference	

*BAs* bronchial arteries, *CI* confidence interval, *HR* hazard ratio, *NBSAs* nonbronchial systemic arteries, *SPSs* systemic artery–pulmonary circulation shunts, *PVA* polyvinyl alcohol

## Discussion

The recurrence of noncancer-related hemoptysis after a successful BAE procedure is a troublesome but inevitable issue in clinical practice. In addition to some definite predictors, the presence of SPS has been inferred to be a potential risk factor for recurrence, but this remains controversial and needs to be further studied [2, 3, 6, 8, 9, 11, 14, 16, 24]. In the present study, the hemoptysis-free survival rates were significantly lower in the SPS-present group than in the SPS-absent group. After adjusting for confounding factors in four statistical models, the results demonstrated high consistency and confirmed that the presence of SPS increased the probability of noncancer-related hemoptysis recurrence after BAE.

Under normal conditions, there is communication between the systemic and pulmonary circulation at both the capillary and precapillary levels, but radiological observation of this is exceedingly rare [12]. In a long-term chronic inflammatory environment, impairment of the pulmonary circulation and collateral pulmonary interstitial hypoxia occur. More systemic arteries are recruited and remodeled, exhibiting both enlargement and angiogenesis to compensate for decreased lung perfusion [25]. Under systemic circulation pressure, the vascular network gradually enlarges to form a pathological SPS, which can then be identified on angiograms. This can also explain the higher number of patients with bronchiectasis and tuberculosis sequela, longer duration of hemoptysis, higher number of affected lobes, larger diameter of culprit arteries, and higher incidence of pleural thickening, lung destruction and culprit NBSAs in the SPS-present group.

In this study, different Cox proportional hazards regression models revealed that the impact of SPSs on recurrence was robust (all P values < 0.05 and HRs > 1). The hemoptysis-free survival rates of the SPS-present group were evidently inferior to those of the SPS-absent group. The majority (38/51) of patients in the SPS-present group experienced recurrence in the first 2 years. More patients in the SPS-preset group than in the SPS-absent group who experienced poor outcomes, including the need for segmentectomy and death due to recurrent hemoptysis. Angiograms of repeated BAE revealed that the rate of recanalization in the SPS-present group was higher than that in the SPS-absent group (13/24 vs. 1/8). This result suggested that culprit arteries complicated by SPS might be prone to recanalization, which was in line with a previous study [3]. The cause of this phenomenon could be the escape and dislocation of embolized materials, which can be induced by the enlargement or rupture of SPSs under systemic pressure and aggravation of local inflammation. As another cause of later recurrence in our study, new collateral artery formation was usually induced by the progression of underlying lung disease. Therefore, the choice of embolic materials for SPSs and gaining control of underlying lung diseases warrant further prospective studies. In addition, the presence of an SPS could potentially increase the risk of nontargeted embolization, such as pulmonary infarction or systemic artery embolization [26]. In our study, one patient in the SPS-present group suffered cerebral lacunar infarction after embolization, which also needs to be considered.

Visualizing SPSs and considering possible outcomes before the BAE procedure seem to be key issues. CTA has the potential to show radiographic indications of SPS in the transpleural systemic arterial supply [27]. Clinically, because CT scans are still suboptimal for identifying the existence and dynamics of SPS, these shunts are mainly identified by angiography. Recently, Qu et al. reported that SPS could be evaluated by dual-input computed tomography perfusion (DI-CTP) in tuberculosis-related hemoptysis patients [28]. However, the radiation exposure rate of DI-CTP is higher than that of conventional CTA. The sensitivity and specificity of DI-CTP and angiography of BAE need to be further compared [28]. Therefore, the possible imaging findings associated with the presence of SPSs need to be further investigated.

This study had some limitations. First, due to its retrospective design, this study has inherent limitations. There were differences in some of the baseline characteristics between the two groups. However, four different Cox proportional hazards regression models were applied to confirm the stability of our hypothesis. Second, although we distinguished between the types of shunts, the type and level of SPSs were hard to stratify in subsequent analysis, which may need further exploration. Third, we did not collect and analyze variables about the inflammation index or treatment for underlying lung disease, which may influence the recurrence rate. However, patients in our study underwent programmed treatment for underlying lung disease during hospitalization and follow-up time.

In conclusion, our study confirmed that the presence of SPS increased the recurrence probability of noncancer-related hemoptysis after BAE. Further treatment strategies for SPSs among hemoptysis patients warrant further prospective studies.

## Data Availability

The data that support the findings of this study are available from the corresponding author upon reasonable request.

## Abbreviations

BAE: Bronchial arterial embolization
CI: Confidence interval
CT: Computed tomography
CTA: Computed tomography angiography
DSA: Digital subtraction angiography
DI-CTP: Dual-input computed tomography perfusion
HR: Hazard ratio
NBSAs: Nonbronchial systemic arteries
PVA: Polyvinyl alcohol
SD: Standard deviation
SPS: Systemic artery–pulmonary circulation shunts
